# Discrete element modeling of a mining-induced rock slide

**DOI:** 10.1186/s40064-016-3305-z

**Published:** 2016-09-21

**Authors:** JianJun Zhao, JianGuo Xiao, Min Lee Lee, YunTao Ma

**Affiliations:** 1 State Key Laboratory of Geohazard Prevention and Geoenvironment Protection, Chengdu University of Technology, Chengdu, China; 2 Chengdu Geological Environmental Monitoring Station, Chengdu, China; 3 Department of Civil Engineering, Lee Kong Chian Faculty of Engineering and Science, Universiti Tunku Abdul Rahman, Kuala Lumpur, Malaysia

**Keywords:** Mining induced rockslide, Crack, Failure mechanism, Discrete element method, Run-out behaviours

## Abstract

Slopes are subjected to stress redistributions during underground mining activities, and this may eventually cause deformation or landslide. This paper takes Madaling landslide in Guizhou Province, China as a case study to investigate the failure mechanism and its run-out behaviours by using discrete element method. Previous qualitative analysis indicated that the slope experienced four stages of failure mechanisms: (1) development of tension cracks, (2) development of stepped-like creep cracks, (3) development of potential rupture surfaces, and (4) occurrence of the landslide. PFC2D program was employed to model the pre-failure deformation characteristics in order to verify the failure mechanisms quantitatively. Subsequently, the run-out behaviours of the landslide were analyzed by PFC3D program. The results indicated that the movement could be summarized into four stages: acceleration stage, constant movement stage, rapid movement stage, and deceleration and deposition stage.

## Background

Mining-induced landslide is a man-made geohazard that has drawn increasing public attention. Studies have shown that these landslides are generally triggered by a confluence of factors including underground mining, topographical and geological conditions (Sun et al. 2007). The underground mining activities may alter the stability of a slope by (1) generating cracks on the slope, (2) causing instability to the slope body through extractions of the slope mass, (3) altering the stress field and geological features of the slope, and (4) altering the subsurface hydrological regime of the slope (Tang 2009).

Longwall mining is a common mining technique used in China. An increase in mining depth may result in a large cavity area known as goaf. The roof above the coal seam is allowed to collapse freely into the goaf upon extractions of the coal. This consequently results in an alteration to the stress state of the slope body, a sagging and bending of the overlying rock strata, a formation of tension cracks, and a subsidence of the ground surface.

It is important to develop a good understanding of the effects of goaf area on the overall stability of a slope. Current trends of studies in mining-induced landslides focus on the development of advanced computing tools such as finite element method and discrete element method (Stead and Eberhardt 1997; Eberhardt et al. 2004; Jiao et al. 2013; Yang et al. 2015; Zhang et al. 2015). In particular, the discrete element method that based on the assumption of earth as discontinuous bodies has proven as an effective method for simulating the deformation propagations of slopes (Li et al. 2013).

Particle flow code (PFC) is a popular discrete element modeling tool derived based on micromechanical properties of granular medium. PFC was first developed by Cundall and Strack (1979) based on assumptions that the micro-particles of rocks or soil materials can be represented by individual spherical particles, while the inter-particle bonds are represented by elastic springs and viscous dampers. Potyondy and Cundall (2004) improved the existing PFC technology for applications specifically in the rock deformation simulation. They successfully simulated the micro-crack generation in rock by altering the inter-particle bonds, while the macro-crack was composed of a series of micro-cracks. This improved model was capable of simulating numerous properties and behaviours of rocks, including the elastic deformation, propagation of cracks, acoustic emission phenomenon, development of anisotropic joints, dilatancy effect, softening effect after the peak stress, and the mechanism of increasing shear strengths with confining pressures etc.

Potyondy and Cundall (2004) successfully performed 2-dimensional and 3-dimensional simulations on granite specimens subjected to compressive stresses and subsequently verified with experimental results obtained from the Brazilian test. Cho et al. (2007) further improved the bonding model of PFC to simulate crystalline rocks. In comparison with another well-known discrete element tool known as Universal Distinct Element Code (UDEC), PFC is widely recognized for its ability to better simulate the destructive behaviours in a rock block. The applications of PFC in rockslide simulations have been proven successful through a series of previous case studies, i.e. Okura et al. (2000), Pirulli et al. (2003), Staron (2008), Poisel and Preh (2008), Tang et al. (2009), Salciarini et al. (2010), Thompson et al. (2010) and Lo et al. (2011).

Sellmeier and Thuro (2011) studied the limitations of using 2-dimensional and 3-dimensional models for rockfall simulations. Modeling of fragmentation scenarios of rockfall remains a significant challenge in rockfall simulations. Preh and Poisel (2007) used PFC3D to back analyze run-out distances of Lärchberg–Galgenwald rockfall. The detached rock mass was modelled as an irregular assembly of particles. Numerical drop tests and back analyses of several rock mass falls were carried out to obtain appropriate damping factors. They concluded that a realistic prognosis of run-outs required a proper calibration of the associated run-out model parameters through back calculations. Recent developments in numerical simulations of rockslide behaviours have seen the use of an advanced numerical technique known as combined finite-discrete element method (FDEM) (Barla et al. 2012). The FDEM offers advantages of simulating progressive development to fragmentation of rock mass and rock avalanche debris.

This study aims to give insights into the failure mechanism and failure propagation of a mining-induced rockslide, known as Madaling landslide 2006. Wang et al. (2013) has presented the interesting failure mechanism and run-out behaviours of the case study based on qualitative observation. The focus of the present study is on discrete element analysis to simulate and verify the pre-failure and post failure deformations of the rock slope with the intention of giving a more insightful understanding on the development of the failure. Firstly, rock specimens are subjected to mechanical properties tests. Secondly, a back analysis is performed to determine appropriate input parameters for the subsequent analyses. Thirdly, the changes in stress state, geological conditions and deformation of the slope induced by the mining activities are simulated using PFC 2D (Version 3.10). The selection of PFC2D is justified by the plain strain condition of the slope during the mining activities in which the deformation is expected to be predominantly occurred in two directions only, i.e. vertical axis and longitudinal axis. The findings are useful for investigating the failure mechanism of the landslide. Finally, failure propagation of the landslide is studied using PFC 3D (Version 3.10). PFC3D is required because the slope terrain and travelling channel were not uniform in the latitudinal direction. For this studied case, PFC is a simpler application of discrete element modeling than UDEC as it adopts rigid disks to simplify the contact detection between rock elements for faster modeling solutions. The rock mass is represented by constituent particles whose contact stiffness and bounding behaviours are relatively simple. In addition, PFC2D allows the breaking-up of blocks to simulate the development of cracks induced by mining activities. These features make PFC 2D a preferred modeling tool used in the present case study than the UDEC.

## Background of study area

### Geographical location

**Fig. 1:**
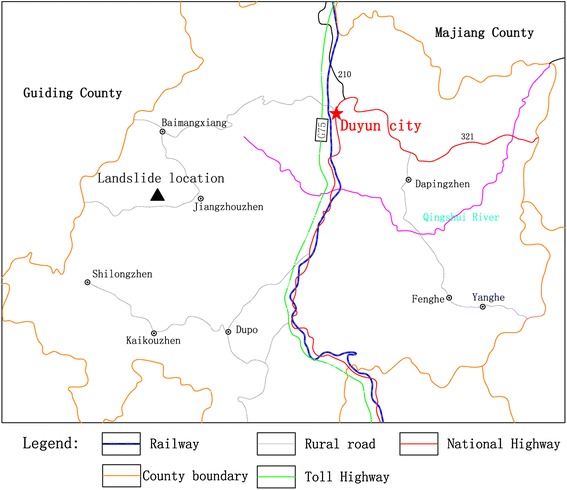
Location map of the study area

The study area is located in Fuxi Village, Guizhou Province, China which is about 22 and 82 km away from Duyun city and Guiyang city, respectively (Fig. 1). The specific geographical coordinates of the landslide area are: E107° 17′ 30″–107° 18′ 28″; N26° 10′ 35″–26° 11′ 29″.

### Topographic conditions

**Fig. 2:**
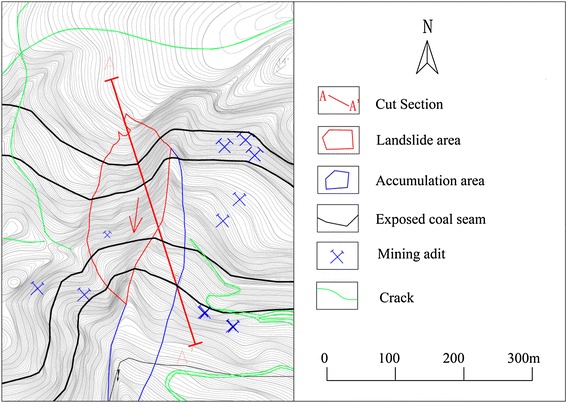
Topography map of the landslide area

Figure 2 shows the topography map of the landslide area. The study site is located in a hilly area with elevations ranging from 1037 to 1595 m. The area has experienced numerous crustal movement and tectonic activities resulting in complex topographical and geological structures. The ground in the northwest region is generally higher than that in the southeast region. The gradient of the slope before failure is ranging from 40° to 60°.

### Geological settings

The study area is located between two North–South trending synclines, namely the Duyun Syncline and the Guiding Syncline. Compressive stress developed along the East–West trending results in numerous faults and joints with strikes of 297° and 072°. The details of the geological settings at the site have been reported by Wang (2004).

### Rock stratification

**Fig. 3:**
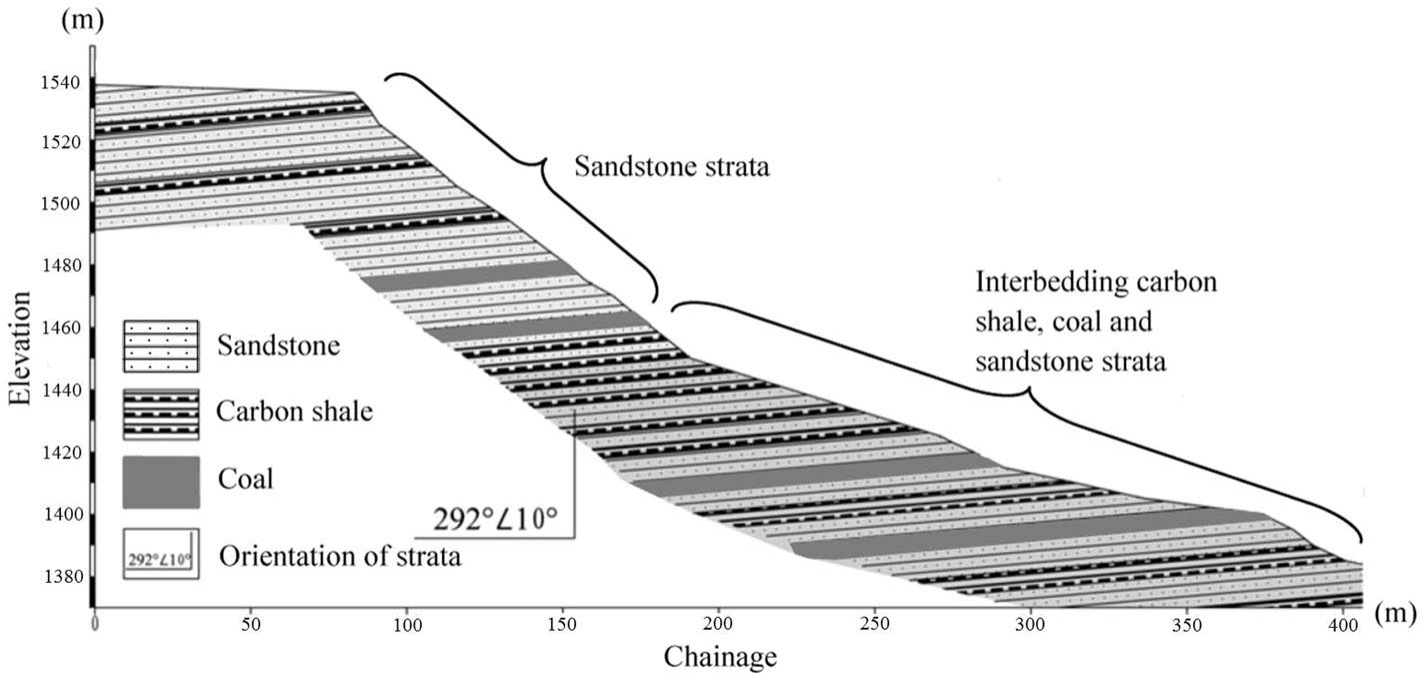
Simplified rock stratifications of the studied slope

The Madaling landslide has a crest elevation of 1540 m and a toe elevation of 1390 m, forming a sloping height of about 150 m. From site investigations, the studied slope generally consists of three rock strata, namely sandstone strata, interbedding carbon shale, coal, and sandstone strata, and limestone strata (Fig. 3). The sandstone (relatively hard rock) strata mainly outcrop at the crest of the slope. Several thin carbon shale and coal layers are found bedded between the sandstone strata. Joint mapping indicates that three sets of discontinuities have been developed in the sandstone strata. The interbedding carbon shale, coal, and sandstone strata mainly occupy the middle part of the slope. The carbon shale is characterized by soft rocks with crushed rock bands. Twelve coal seams were exposed on the sloping surface. These thin carbon shale and coal layers were intebedded by the relatively hard sandstone layer. The lowest stratum comprises the relatively hard limestone. The limestone stratum is omitted from the subsequent analysis and discussion in this paper because the slip plane of the landslide has not extended into this stratum.

### Human activities

Coal mining activities have been active at the study area since half a century ago. Most of the mining activities were carried out by inclined mining adits. In addition, there were numerous illegal coal mining activities operated by local residents. These small-scaled illegal mining activities were generally scattered randomly within the study area with an average mining depth of about 200 m. The mining activities undertook very minimum safety measures, whereby most of the goaves were only either supported by a temporary shoring system or even left unsupported.

## Failure mechanism and propagation of Madaling landslide

### Failure mechanism

**Fig. 4:**
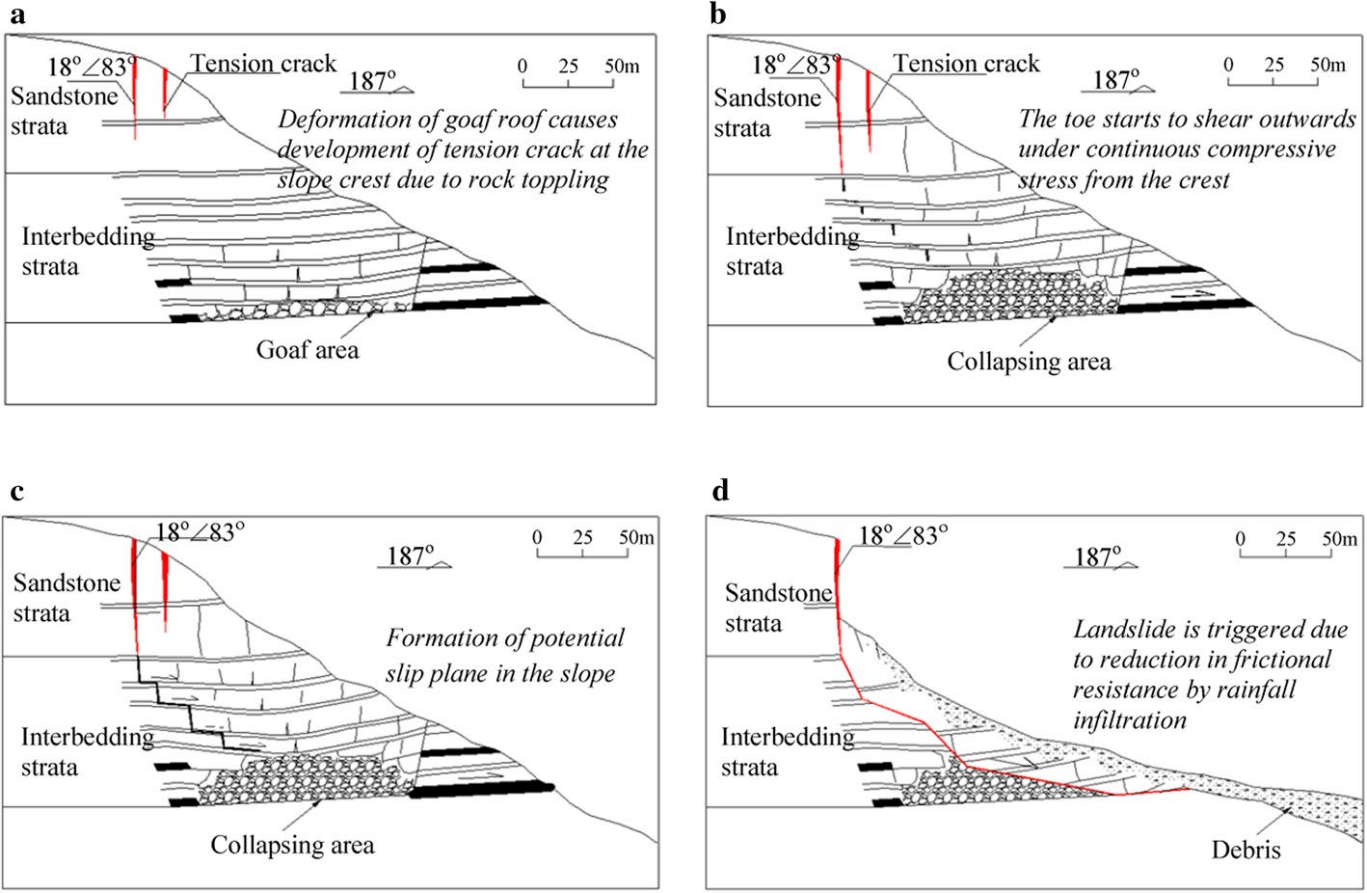
Failure mechanism of the Madaling landslide. (**a**) Development of tension cracks due to collapses of goaf roof, (**b**) Development of stepped-like creep crack due to shear deformation, (**c**) Development of potential slip surface, (**d**) Occurrence of landslide. Modified from Wang et al. (2013)

Based on field data and previous reported studies (Wang et al. 2013), the failure mechanism of the Madaling landslide can be summarized into four processes as shown in Fig. 4.

- Development of tension cracks due to collapses of goaf roof

Illegal mining activities have formed numerous unsupported goaf areas. Collapses of the goaf roof caused the overlying rock strata to settle due to gravitational force. This phenomenon caused the overlying rock mass at the goaf boundary to deform outwards and eventually developed deep tension cracks.

(b) Development of stepped-like creep crack due to shear deformation

A shear deformation was developed in the interbedding soft and hard rock layers as results of compressive stress from the overlying hard rock layer and outward deformation of the rock at the inner part of the slope. Stepped-like creep crack started to develop in the interbedding layers, particularly in the soft rock layer.

(c) Development of potential slip surface

A potential slip surface was formed through connections of the tension cracks developed in the upper hard rock layer, stepped-like creep crack at the middle of the slope, and shear crack at the toe. Rainfall infiltration has further weakened the shear strength of the rock, particularly at the toe area.

(d) Occurrence of landslide

Under prolonged rainfall infiltrations, the frictional resistance along the potential slip surface was weakened by the rainwater. The stepped-like creep crack was connected gradually under intensified stresses. The landslide was finally triggered resulting in the rapid debris flow.

### Failure propagation

**Fig. 5:**
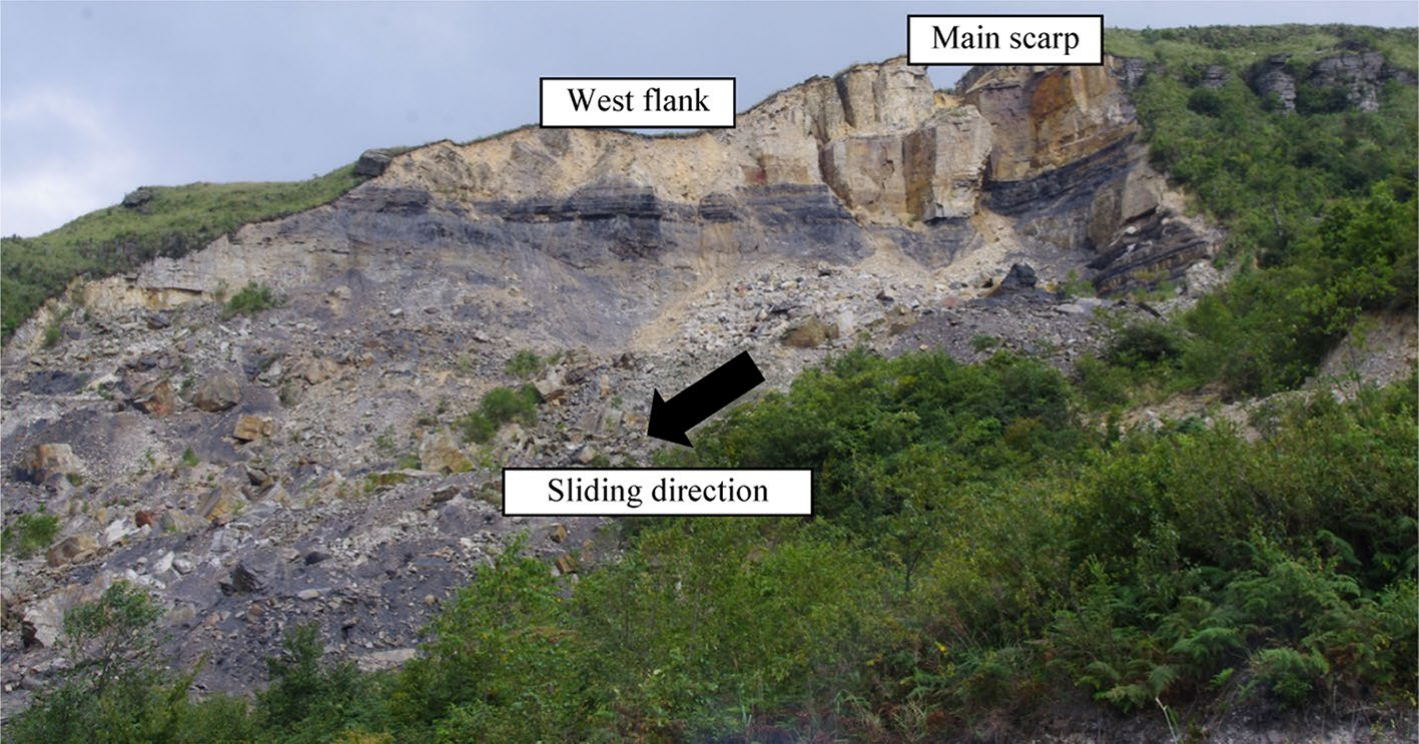
Photograph of the Madaling landslide

The Madaling landslide occurred at 4.00 a.m. on 18th May 2006 generating a sliding mass of approximately 1.9 × 10^6^ m^3^. The landslide has a visible and close-to-vertical west flank and a main scarp in the directions of N56°W and N22°E, respectively (Fig. 5). Two tension cracks were observed at the crest behind the main scarp, while evidences of previous illegal mining activities were spotted at the toe.

From back analysis and field observation, it was found that the rock masses slid at an extremely rapid speed, with the maximum speed deduced as approximately 39 m/s. The velocity of slide was predicted by the following equation (Hungr et al. 2001):

1 $$ V_{s} = \sqrt {1 - \frac{f}{\tan \alpha } - \frac{cl}{W \cdot \sin \alpha }} \cdot \sqrt {2gH} $$

where *V*_*s*_ = velocity of slide, *f* and *c* = parameters associated with resistances developed along the sliding plane, *α* = sliding angle, *W* = weight of sliding mass, *H* = height of sliding mass centroid and *L* = length of sliding mass.

**Fig. 6:**
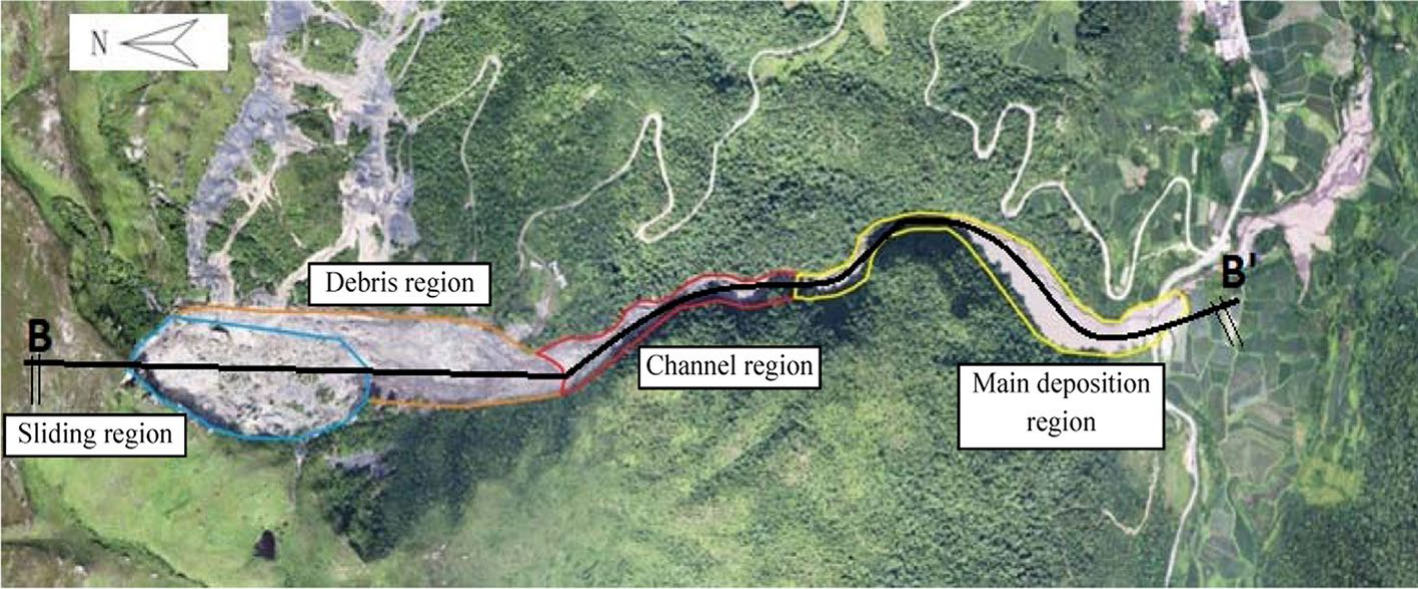
Overview of the landslide and accumulation areas

**Fig. 7:**
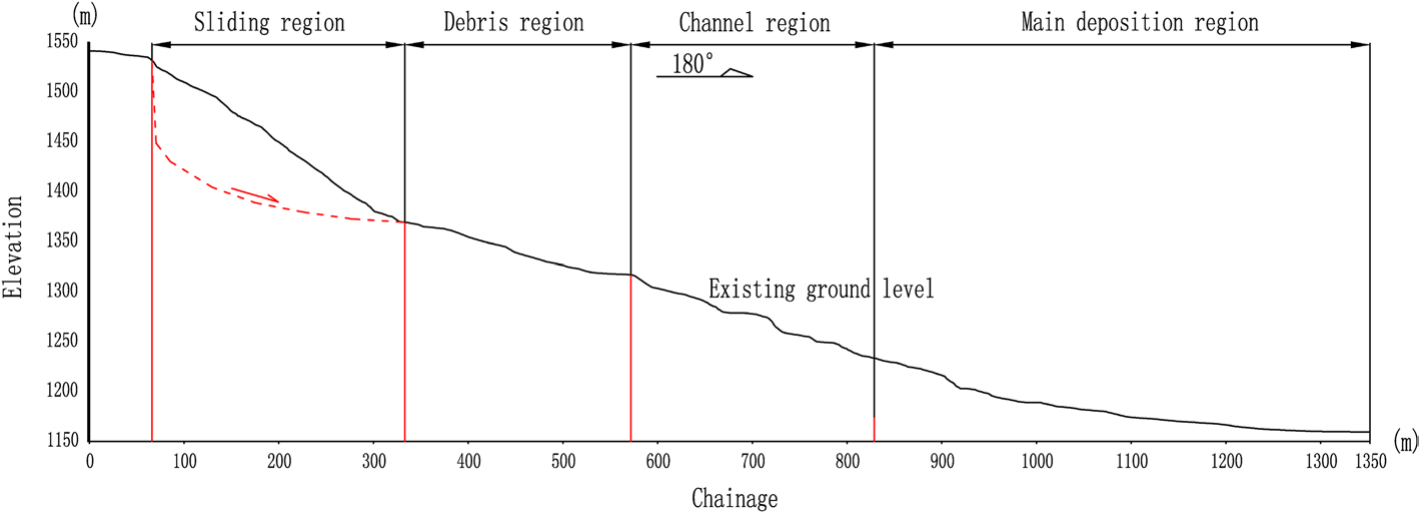
Cross-sectional view of the landslide and accumulation areas

The gravitational movement of the sliding mass caused a huge impact when hitting on a small hill located at the eastern side. Consequently, the rock blocks were broken down into debris of smaller particles. Rainwater acted as a lubricant to facilitate rapid flows of the rock debris. This eventually resulted in a long accumulation area of about 1.5 km covering a large part of the plantation areas at the downslope. Based on the failure propagation and accumulation characteristics, the landslide accumulation can be categorized into four regions, namely sliding region, debris region, channel region, and main deposition region, as shown in Figs. 6 and 7. The sliding region at the elevation of above 1400 m consisted of mainly 1–2.5 m of sandstone blocks which were residues of collapsed rock mass from the flank walls. The debris region at the elevations of 1350–1400 m was formed by disintegration of the sliding rock blocks as the result of huge impact when the rock blocks hit on the hill at the eastern side, as mentioned earlier. Residue rock debris were flowed to the channel region at the elevations between 1250 and 1350 m. Surface water flow and the narrow cross-section in the channel region contributed to a favorable condition for the rapid debris flow. The main deposition area was located at a relatively flat and wide farmland area at the elevation of below 1250 m. The deposition contained mainly sandstone boulders of >63 mm, shale gravels of 2–63 mm, cinder, and a large amount of mud forming a total thickness of 5–8 m.

## Two-dimensional modeling of failure mechanism

The deformation characteristics of the Madaling slope during pre-failure stage were simulated using PFC 2D model to verify the failure mechanism as discussed in the “Failure mechanism” section. The details of the modeling approach and results are provided in the following sub-sections.

### Modeling parameters

**Fig. 8:**
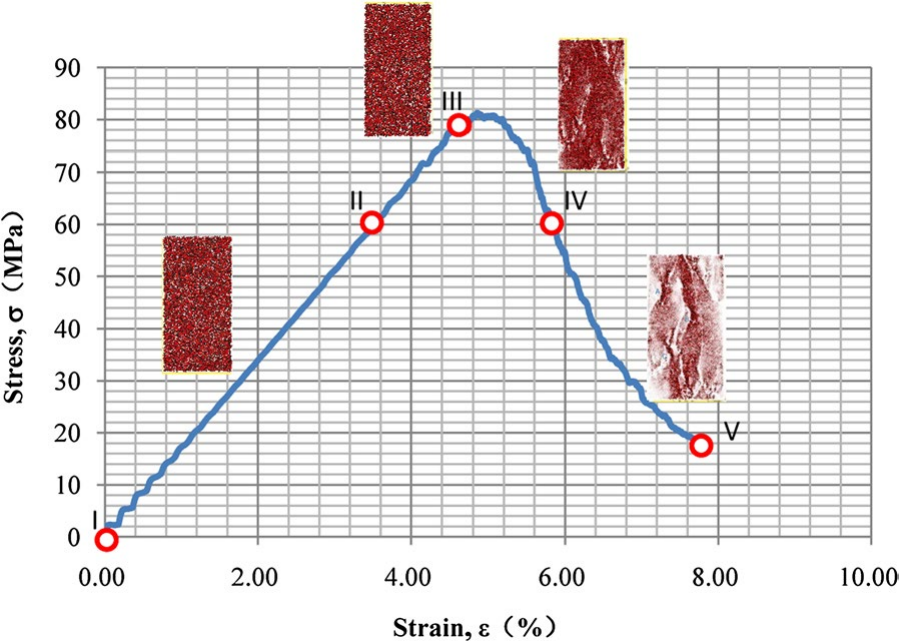
Stress–strain relationships and the simulated development of micro-cracks on the rock specimens at zero confining stress

PFC model requires input parameters of microstructure properties (inter-particle contacts) that are capable of representing macroscopic behaviours of the modeled mass. Therefore, it may not be appropriate to apply the properties obtained from the laboratory tests directly into the PFC model. A series of parametric simulations and laboratory tests were first performed to match the simulation results with the laboratory test results. This calibration process was important to obtain the adequate input parameters for the subsequent modeling of the actual slope. The laboratory tests consisted of the conventional triaxial test and the Brazilian test with specimen sizes of 50 mm in diameter and 100 mm in height, and 50 mm in both diameter and height, respectively. Eight confining pressures ranging from 0 to 100 MPa were applied to the triaxial samples with the resultant peak differential stress of about 70–80 MPa. PFC simulations were performed to match the properties obtained from the laboratory tests. From the laboratory test results, the rock specimens typically underwent five stages of stress–strain characteristics under compressive stresses. Stage I–II corresponds to the linear elastic state. Stage III is the peak strength. Stage IV–V is the residual strength state (Fig. 8). The experimental tests enabled observations of crack development in the rock specimens.

Zhao et al. (2012) studied the effect of micro-properties on macro-properties of rocks using uniaxial compression test and parallel bonded model. The micro-properties refer to properties at the ball/particle scale, while the macro-properties refer to overall properties at the specimen scale. They found that when the ratio of sample size to particle size is larger than 100, the effect of particle size on Poisson ratio (*v*) is negligible. Therefore, the particles of PFC model should be sufficiently fine to minimize their effects on the Poisson’s ratio. In addition, Zhao et al. (2012) also reported that the particle size does not have any significant effect on the Young’s modulus (*E*). The values of *v* and *E* are functions of the parallel bond stiffness ratio (*k*_*n*_*/k*_*s*_):

2 $$ E/E_{c} \, = \, - 0.27\ln \left( {k_{n} /k_{s} } \right) + 1.36\quad \left( {{\text{r}}^{2} = \, 0.952} \right) $$

3 $$ v = 0.12\ln \left( {k_{n} /k_{s} } \right) + 0.2\quad \left( {{\text{r}}^{2} = \, 0.855} \right) $$

where *E*_*c*_ is the elastic contact modulus of particles, *k*_*n*_ is the normal stiffness and *k*_*s*_ is the tangential stiffness. Zhou et al. (2012) investigated the effect of micro-properties on the friction angle (*ϕ*) and cohesion (*c*) of macro model. They proposed the following empirical equations:

4 $$ c/BS_{S} = 73.76 + 19.51\ln \left( K \right) + 18.38\ln \left( {k_{n} /k_{s} } \right)\quad \left( {{\text{r}}^{2} = \, 0.961} \right) $$

5 $$ \phi = 24.56 - 0.21\,\left( {k_{n} /k_{s} } \right) + 6.15\ln (\mu )\quad \left( {{\text{r}}^{2} = \, 0.995} \right) $$

where *K* = *BS*_*N*_*/BS*_*S*_, *BS*_*N*_ is the normal bond strength, *BS*_*S*_ is the shear bond strength, *μ* is the coefficient of friction between particles.

The parametric simulation adopted initial input properties derived from the Eqs. 2–5. The modeled particle sizes ranged between 0.3 and 1.5 mm. Modenese (2013) highlighted the importance of selecting a representative elementary volume for a DEM model in which the number of grains is adequate to eliminate any adverse impact on the numerical results. Prior to simulations of the actual slope, a sensitivity analysis was performed to study the effect of model size ratio on the result of simulation based on the triaxial specimen size. The specimen width to particle diameter ratio (*l/d*) of about 160 was found to be adequate for both the PFC2D and PFC3D models. From the results of parametric simulations and laboratory tests, the modeling parameters for the subsequent PFC analysis were calibrated and summarized in Table 1.

**Table 1:** Input parameters for PFC2D model

Parameters	Sandstone	Carbon shale	Coal
Model height (mm)	100		
Model width (mm)	50		
Particle size (mm)	0.55 (diameter)		
Number of particles	17,900		
Total time step	6 × 10^4^		
Porosity (%)	14	14	14
Particle density (kg/m^3^)	3000	2700	2400
Particle friction coefficient (μ)	0.38	0.75	0.62
Bond radius ratio (λ)	1	1	1
Normal contact stiffness (GPa)	18	8.3	0.5
Shear contact stiffness (GPa)	13	4.4	0.45
Parallel bond normal stiffness (GPa)	5.3	2.4	0.26
Parallel bond shear stiffness (GPa)	3.8	1.3	0.23
Normal bond strength (MPa)	50	11	3.27
Shear bond strength (MPa)	50	11	3.27

### PFC2D model for pre-failure deformation analysis

**Fig. 9:**
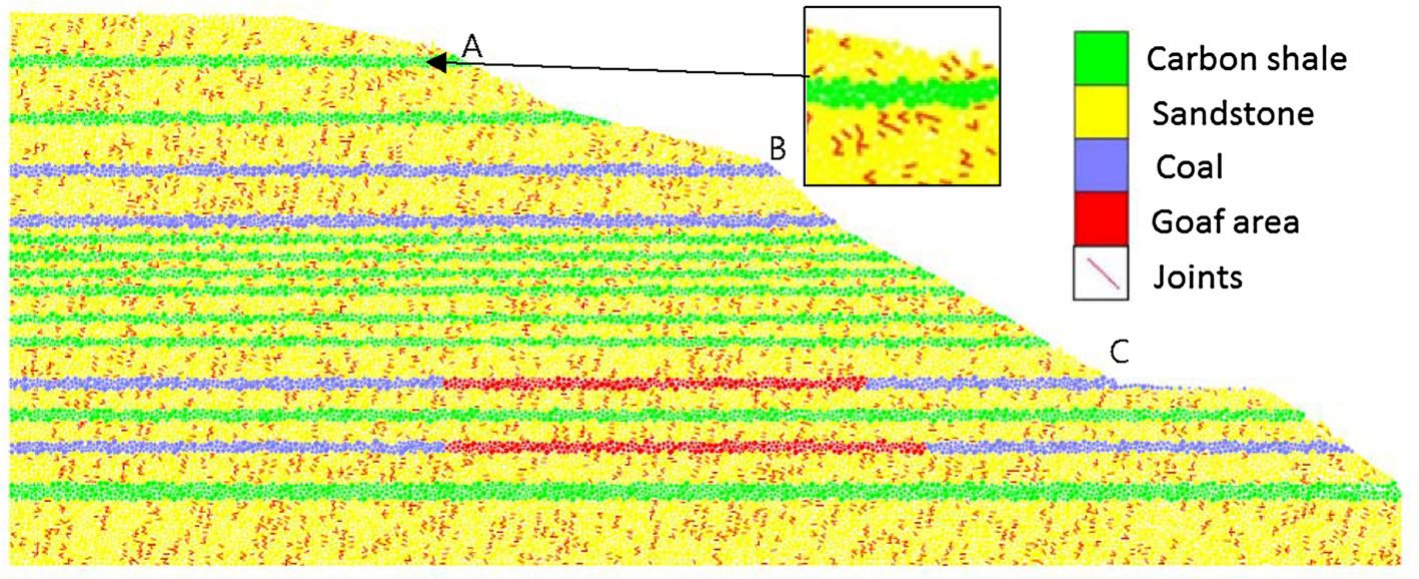
Simulated PFC2D model

Figure 9 shows the simulated PFC2D model of the Madaling slope comprising sandstone layer, carbon shale layer, coal layer and goaf area. The horizontal beds of the rock strata were simplifications of the actual profile that consisted of strata inclined at 10°. The model, 190 m in height and 492 m in length, was formed by 25,000 particles. Table 1 tabulates the input mechanical properties of the model. The geometrical characteristics of joints were simulated by referring to the study reported by Wang (2004). Considering most of the joint interfaces were relatively smooth and the gaps were not in-filled, the bond strength and joint friction coefficient were assumed to be negligible. The side and bottom boundaries of the model were fixed and a stress/strain controlled deformation was applied. No tectonic stress was considered in the analysis.

A steady state was first achieved by applying an initial stress field (gravitational field) to generate a heterogeneous stress field. The goaf areas were then removed instantaneously to simulate the start of the mining activities. This time step marked the start of the time-dependent deformation analysis. The analysis was halted at the time step of 1 million when the static equilibrium condition was achieved. 1 time step represented 1 s. PFC by default applies a local and non-viscous damping proportional to acceleration of movement for every individual particle. The damping force is controlled by a constant with a default value of 0.7. This damping model is best suited to a quick calculation of equilibrium. However, this will cause the disadvantage of the particle movements being damped as well. Therefore, no numerical damping was adopted in this analysis with the assumptions that energy dissipations during the landslide were only derived from the frictional forces between particles.

### Results of simulated pre-failure deformation

**Fig. 10:**
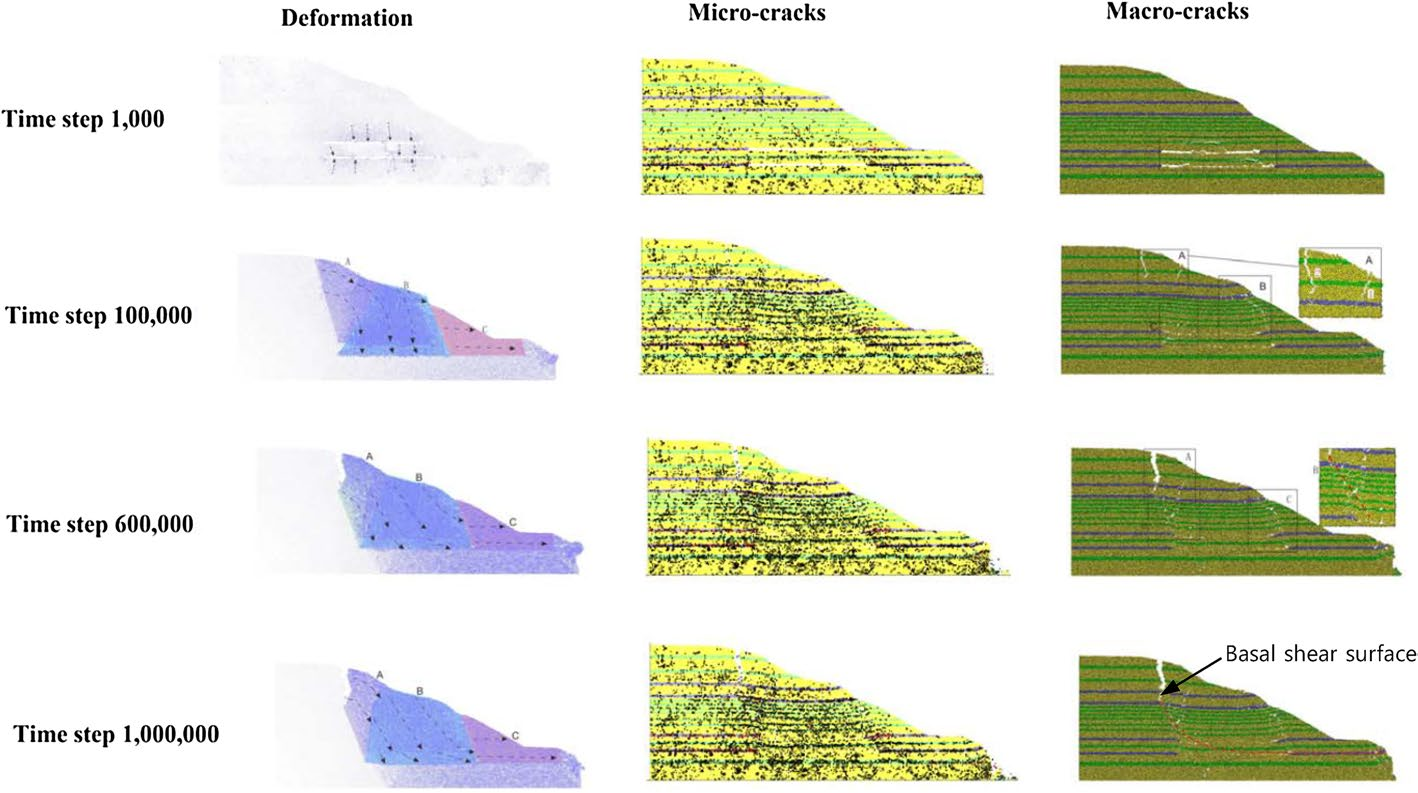
Development of deformation, micro-cracks, frictional and cohesive stresses in the simulated slope

Figure 10 presents the summary of the analysis results at selected time steps (time steps 1000, 100,000, 600,000, and 1,000,000) encompassing development of deformation, micro-cracks, and macro-cracks in the rock slope. Micro-cracks refer to cracks isolated from the neighbouring cracks while macro-cracks are inter-connected network of cracks.

1. Time step 1000

This time step was selected to show the deformation in slope immediately after the completion of mining activities. Due to the unsupported mining activity, the goaf roof experienced a localized settlement as large as 1.8 m. Stresses mainly developed at 25 m below the slope surface, indicating that the slope was susceptible to a shear failure. In addition, most of the tensile stresses focused at the goaf roof and the crest of the slope.

(2) Time step 100,000

The goaf roof experienced a complete collapse at this time step. The slope can be divided into three compartments, namely (A) Toppling zone, (B) Settlement zone, and (C) Sliding zone. The gradual settlement in zone B provided a large space for toppling to occur in Zone A. Two tension cracks were developed at the crest: Tension crack 1 with a width and a depth of 0.8 and 18 m, respectively; and tension crack 2 with a width and a depth of 2.4 and 24 m, respectively. Unfortunately, the actual field crack width cannot be traced as the field investigation was only carried out after the rockslide occurrence. From the field investigation, five tension cracks with widths of 1.0–9.5 m were observed on a neighbouring slope which was located at about 400 m away from the rockslide site. In zone C, the slope only deformed laterally for about 0.15 m.

Despite of the fact that the elapsed time at this time step was only 10 % of the total analysis steps, most of the fractures and cracks were developed within this period. There were a total of 6400 (84.21 %) fractures/cracks detected in the slope, in which 96.88 % of the cracks were formed under tensile stress. The shear cracks mainly focused at the toe of the slope.

Stresses mainly developed at the crest and the side boundary of the collapsing goaf roof areas, indicating that the corresponding area was subjected to shear deformation. Less stresses were developed at the goaf area considering the mass has settled considerably and the structures have been destroyed.

(3) Time step 600,000

At this stage, most of the microscopic fractures were inter-connected forming a continuous internal discontinuity. The tension crack near the slope crest has been enlarged to 5 m wide and developed to the depth of the coal layer. Eventually, the deep tension crack and the internal discontinuity were connected forming a potential slip plane. At this stage, the development of new fractures showed a decreasing rate.

The main difference of the deformation characteristics between the time steps 100,000 and 600,000 was that the crushed mass in Part B of the time step 600,000 tended to deform outwards under increasing compressive stress from Part A. The Part A, in turn, experienced more severe toppling as the result of the outward deformation of the Part B. There were, however, no notable changes in stresses in the slope compared to the previous stage.

(4) Time step 1,000,000

This is the last step of the analysis indicating the end of the slope deformation prior to landslide occurrence. At this stage, there was no new fracture generated in the slope. Under increasing compressive stress from the Part A, the mass in part B has crept further, while the mass at the bottom of part C experienced shear deformation. This phenomenon resulted in a complete development of a potential slip plane initiated from the crest of the slope and extended until the toe of the slope. It was expected that the failure started to take place after this time step.

## Three-dimensional modeling of failure propagation

A three-dimensional PFC model was employed to simulate the failure propagation of the Madaling landslide. Firstly, a three-dimensional model was used to back analyze a 10 cm × 5 cm × 5 cm (height × length × width) rectangular specimen to obtain the required input parameters for the subsequent analysis. The mechanical properties of the associated rock mass for the three-dimensional modeling are summarized in Table 2. The modeling parameters adopted in the PFC3D model (Table 2) were different from that of PFC2D (Table 1). These modeling parameters were obtained from the back analysis on laboratory testing results on triaxial specimens of 100 mm height × 50 mm diameter using PFC2D and PFC3D, respectively. In general, the parameters obtained from the PFC3D analysis yielded a higher bonding strength than those of PFC2D because of considerations of the more rigid 3-dimensional bonds.

**Table 2:** Input parameters for PFC3D model

Parameters	Sandstone	Carbon shale	Coal
Model height (mm)	100		
Model width (mm)	50		
Particle size (mm)	1.09 (diameter)		
Number of particles	39,086		
Total time step	1.3 × 10^5^		
Porosity (%)	15	15	15
Particle density (kg/m^3^)	3000	2700	2400
Particle friction coefficient (μ)	0.38	0.75	0.62
Bond radius ratio (λ)	1	1	1
Normal contact stiffness (GPa)	11.8	4.2	0.5
Shear contact stiffness (GPa)	8.44	2.3	0.5
Parallel bond normal stiffness (GPa)	5.3	2.4	1.0
Parallel bond shear stiffness (GPa)	4.0	1.3	0.5
Normal bond strength (MPa)	80	10	5
Shear bond strength (MPa)	80	10	5

It should be noted that the actual mechanical properties of the slope mass should be lower than those obtained from laboratory tests considering the structures of the slope mass have been altered by the mining activities prior to the landslide occurrence. Therefore, it was appropriate to multiply a reduction factor (η) to all the mechanical properties obtained. By comparing the simulated and actual characteristics and position of the accumulation after the landslide propagation, the reduction factor as identified from the back analysis was 0.3. This reduction factor may differ in other analyses with different geomechanics and material conditions.

**Fig. 11:**
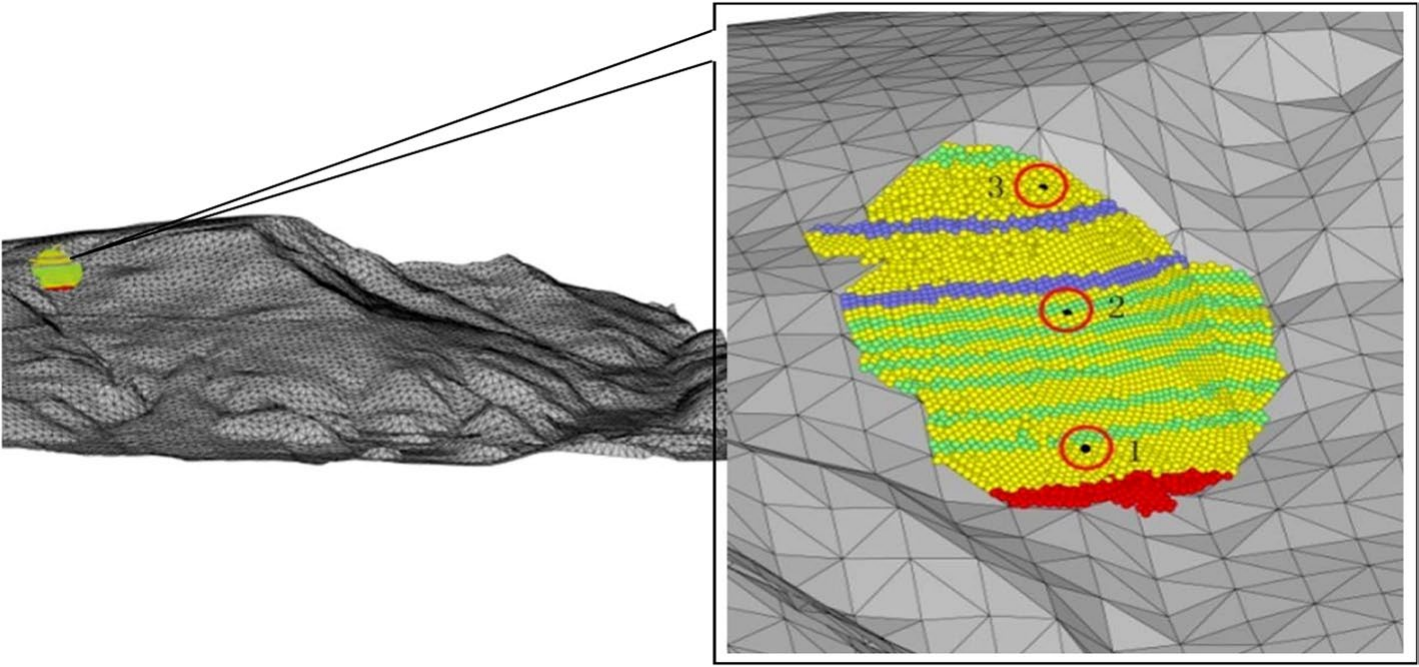
Simulated PFC3D model

Figure 11 shows the topography of the study area encompassing an area of 2100 m × 1600 m. The boundary between the topography and the sliding mass was modeled as a frictional boundary with the friction angle was assumed to be close to the angle of repose of the natural materials, i.e. 30°. The landslide affected area with a total sliding volume of 1.9 × 10^6^ m^3^ was modeled by 21,564 particles. Simulations with finer particles would enhance the quality of the analysis but would certainly increase the computational cost as well. It is important to select an appropriate fineness level of the simulation while not compromising the reliability of the findings obtained. The number of particles modeled in this study was comparable to the previous work reported by Lu et al. (2014) who employed 30,000 and 35,000 particles for sliding volumes of 1 × 10^7^ and 1.3 × 10^7^ m^3^, respectively. To facilitate the observation of mass movement, three monitoring points, namely points 1, 2, and 3 were indicated at the toe, middle and crest of the slope, respectively.

**Fig. 12:**
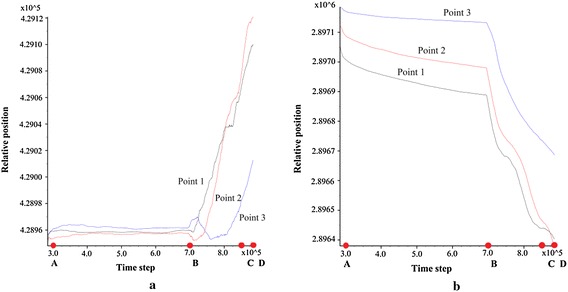
Deformation–time relationships in **a** X-direction, **b** Y-direction. *Note* 1 time step = 1 s

**Fig. 13:**
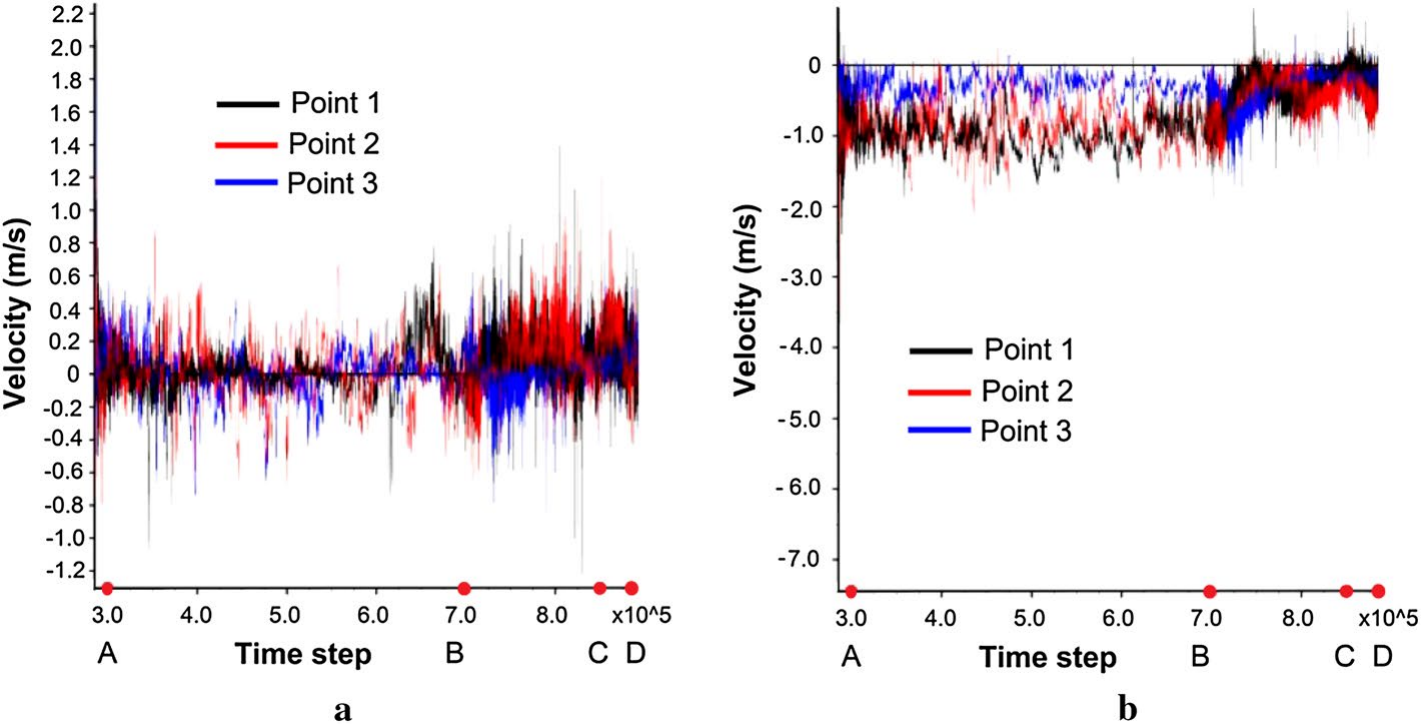
Velocity–time relationships in **a** X-direction, **b** Y-direction. *Note*: 1 time step = 1 s

From the deformation-time graphs (Fig. 12), the slope has experienced four stages of mass movement: acceleration stage (before point A), constant movement stage (between AB), rapid movement stage (between BC), and deceleration and deposition stage (between CD). These results were consistent with the velocity–time relationship shown in Fig. 13. In addition, the deformation-time graph also indicated that the mass at the monitoring point 2 has travelled longer than the mass at points 1 and 3 at the end of the landslide movement. The results implied that the mass at the middle of the slope has travelled at the highest velocity compared to those at the toe and crest. The velocity–time graph exhibited a fluctuating pattern indicating that the velocity of the mass movement was inconsistent due to collision between the fragmented rocks.

**Fig. 14:**
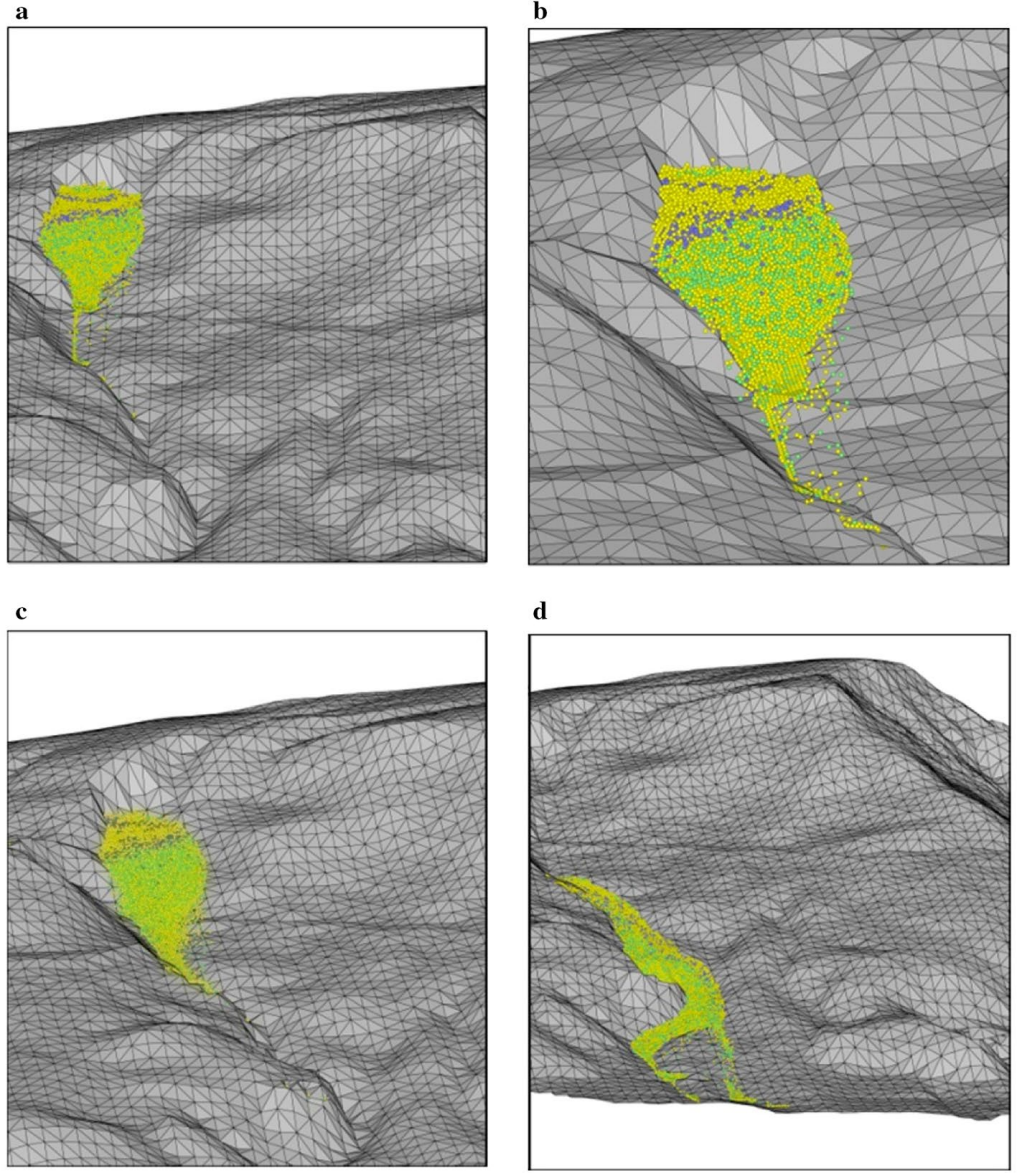
Stages of simulated landslide propagation: **a** sliding, **b** deceleration at toe, **c** re-acceleration when debris entering the narrow channel, **d** deposition at the channel outlet

**Fig. 15:**
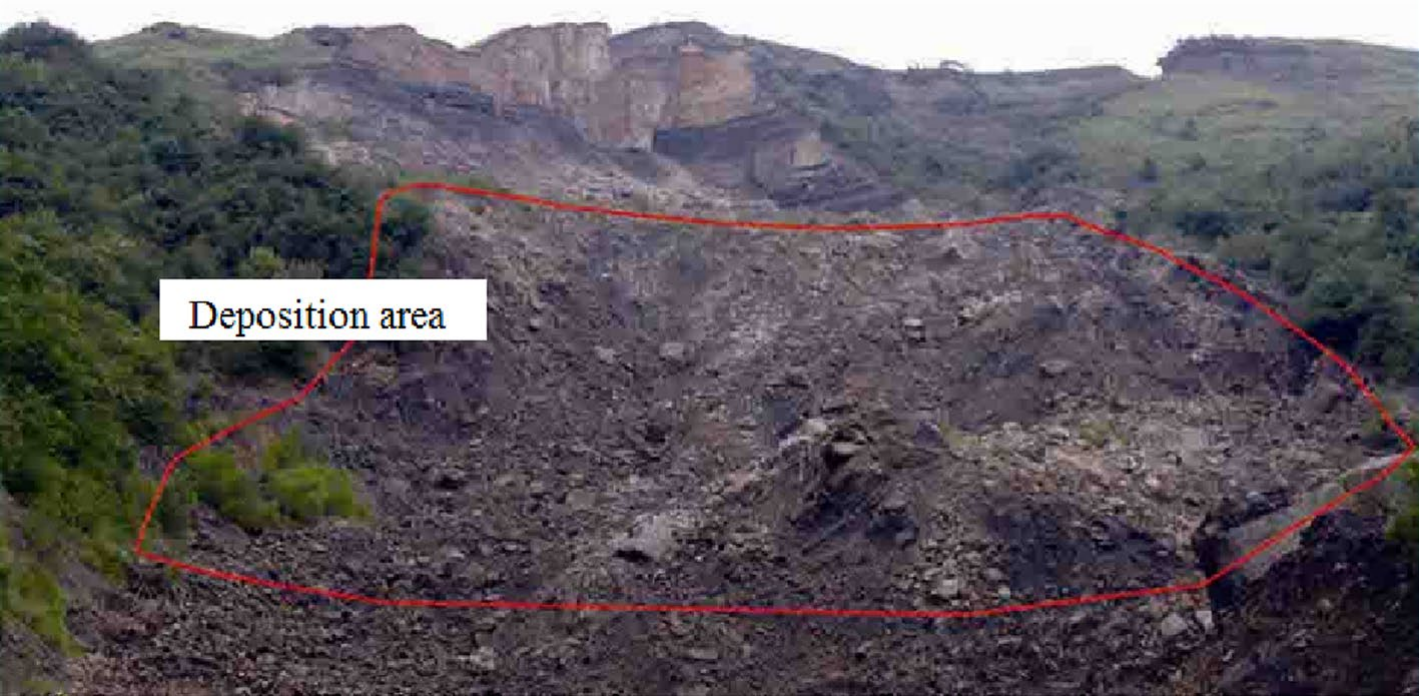
Photograph of deposition at the channel outlet

The sliding mass has experienced different run-out behaviours during the four prescribed stages of mass movement. During the acceleration stage which occurred within the first 100,000 time steps, the maximum velocity has reached 2.5 m/s. The mass deformed rapidly through a shearing along the sliding plane (Fig. 14a). The sliding mass was then deposited on a relatively gentle toe and experienced a relatively slow movement (Fig. 14b). The collision between sliding mass resulted in fragmented particles and eventually formed a debris flow. The debris flow was subsequently travelled into a narrow and steep channel which re-accelerated the mass to travel at a high velocity (Fig. 14c). The wide and gentle topography coupled with the bending nature of the channel near the outlet caused the debris flow to decelerate and eventually deposited on the channel outlet (Fig. 14d). The longest run-out distance obtained from the analysis was 260 m, which was consistent with the actual field observation (Figs. 6 and 15).

The Madaling slope was not instrumented before and during the landslide occurrence. Therefore, direct validations of the simulated run-out behaviours become practically impossible. These limitations have restricted the possibility of performing quantitative comparisons between the numerical simulation results and the field data. Nonetheless, the computations of important quantities such as run-out velocity and distance, as well as thickness of deposition can still be verified from the post-failure field investigation. These indirect validations/comparisons could increase the level of confidence towards the validity of the simulated results. Crosta et al. (2003) reported that in situ evidences like major tension or shear cracks, post-event descriptions of the main failure surface and depositions could be useful qualitative evidences to validate the simulation results. Numerous researchers adopted laboratory scaled granular flow experiments or down-scaled slope models to validate the actual run-out behaviours of landslides (Major 1997; Lajeunesse et al. 2006; D’Agostino et al. 2010). These laboratory experiments can be performed in future studies to improve the insightfulness of the investigation.

## Conclusion

This paper presents the results of numerical analyses to provide insights into the failure mechanism and failure propagation of the Madaling landslide 2006. The findings of the present study provide useful quantitative evidences for validating the failure mechanism reported by Wang et al. (2013). Even though the present study generally agreed with the mechanical mechanism reported from the previous study, this study still contributed to several new findings, such as information about the material properties at the time of failure, predicted characteristics of tension cracks developed on site, and ground settlement as the result of the mining activities etc. These information were not retrievable from site investigation as the investigation works were only carried out after the failure.

The Madaling slope was mainly induced by goaf area left unsupported from previous mining activities. Despite of the fact that rainfall has been identified as another triggering factor for the occurrence of the Madaling landslide, the effect of the rainfall on the stability of the slope, however, is beyond the scope of the present study and will be reported in another paper. The present study focuses primarily on the stress state and deformation of the slope induced by the previous mining activities. PFC2D was used to simulate the failure mechanism of the slope. The input parameters of the model were calibrated carefully by performing laboratory experiments on field specimens and back analyses using representative PFC models.

The results of the two-dimensional discrete element analysis (PFC2D) indicated that the goaf area created by previous mining activities has caused the slope to undergo three stages of deformation, i.e. (1) Rapid developments of micro-fractures in the slope as the result of collapses of the goaf roof; (2) Formations of continuous micro-fractures and tension cracks under compressive stress from the toppling zone, (3) A development of shear plane at the toe of the slope forming a complete potential slip plane in the slope. It should be noted that the mined out area was simulated by simply removing the particles at the area concerned to initiate the rock deformation. The collapse of the goaf may occur through many possible mechanisms. These mechanisms, however, was not studied explicitly in the present study. Based on the deformation characteristics, the slope can be divided into three compartments, namely toppling zone, settlement zone, and sliding zone. These zones play an important role in generating the micro-fractures, and subsequently the potential slip plane of the slope.

The failure propagation of the slope was subsequently modeled by a three-dimensional discrete element analysis (PFC3D). A reduction factor of 0.3 was applied to the mechanical properties obtained from the laboratory experiments to account for deteriorations of rock structures caused by the fractures and cracks developed in the slope. In addition, residual stresses were mobilized during the debris flow. The mass movement during the landslide occurrence can be categorized into four stages, namely acceleration stage, constant movement stage, rapid movement stage, and deceleration and deposition stage. Although the simulation results could not explain the mechanism of shear strength reduction of the materials, the simulation successfully provided scenario-based run-out paths, sliding velocity and affected area. These information are useful for assessing the potential landslide hazard of another slope (Jieniangping slope) which is located at about 400 m away from the present failed slope and had shown signs of failures.

In summary, the failure mechanism and failure propagation of the Madaling landslide can be attributed to several factors including mining activities, topography and geological conditions of the slope. The mining activities created unsupported goaf areas that promoted development of fractures and cracks in the slope under compressive stress. The existence of soft rock layers in the slope created favourable conditions for the development of the fractures. During the failure propagation, the hill at the eastern flank acts as a resistant wall that crushed the sliding rock into fragmented particles and direct them into a narrow channel. The narrow channel provides a favourable condition for rapid debris movement. The combinations of these topographic conditions resulted in the long run-out distance as observed in the present study case.
